# Spinal cord involvement and contrast enhancement in posterior reversible encephalopathy syndrome

**DOI:** 10.1259/bjrcr.20150326

**Published:** 2016-10-20

**Authors:** Himanshu Agarwal, Leve Joseph Devarajan Sebastian, Shailesh B Gaikwad, Ajay Garg, Nalini Kant Mishra

**Affiliations:** Department of Neuroradiology, All India Institute of Medical Sciences, New Delhi, India

## Abstract

Although posterior reversible encephalopathy syndrome (PRES) is a widely encountered clinicoradiological entity, spinal cord involvement on MRI is very rarely reported. We found only eight cases that have been reported so far. Reports of post-contrast meningeal or parenchymal enhancement in PRES are even rarer. Herein we report a case of PRES with extensive spinal cord signal abnormality with contrast enhancement. Familiarity with this rare imaging finding of PRES, in the appropriate clinical setting, will avoid unnecessary investigations and inappropriate treatment.

## Summary

Although posterior reversible encephalopathy syndrome (PRES) is a widely encountered clinicoradiological entity, spinal cord involvement on MRI is very rarely reported. We found only eight cases that have been reported so far. Reports of post-contrast meningeal or parenchymal enhancement in PRES are even rarer. Herein we report a case of PRES with extensive spinal cord signal abnormality with contrast enhancement. Familiarity with this rare imaging finding of PRES, in the appropriate clinical setting, will avoid unnecessary investigations and inappropriate treatment.

## Case presentation

A 14-year-old male presented to the emergency department with two episodes of seizure preceded by a history of headache for 1 month and diminution of vision in both eyes for 5 days. His past history was significant for appendicectomy 3 years ago and severe abdominal pain 2 months ago. On examination, the patient was conscious, oriented and comprehending, with no neurological deficits. His blood pressure was 170/110 mmHg and other vitals were normal. A fundoscopic examination later revealed Grade 4 hypertensive retinopathy.

## Investigation

**Figure 1.**
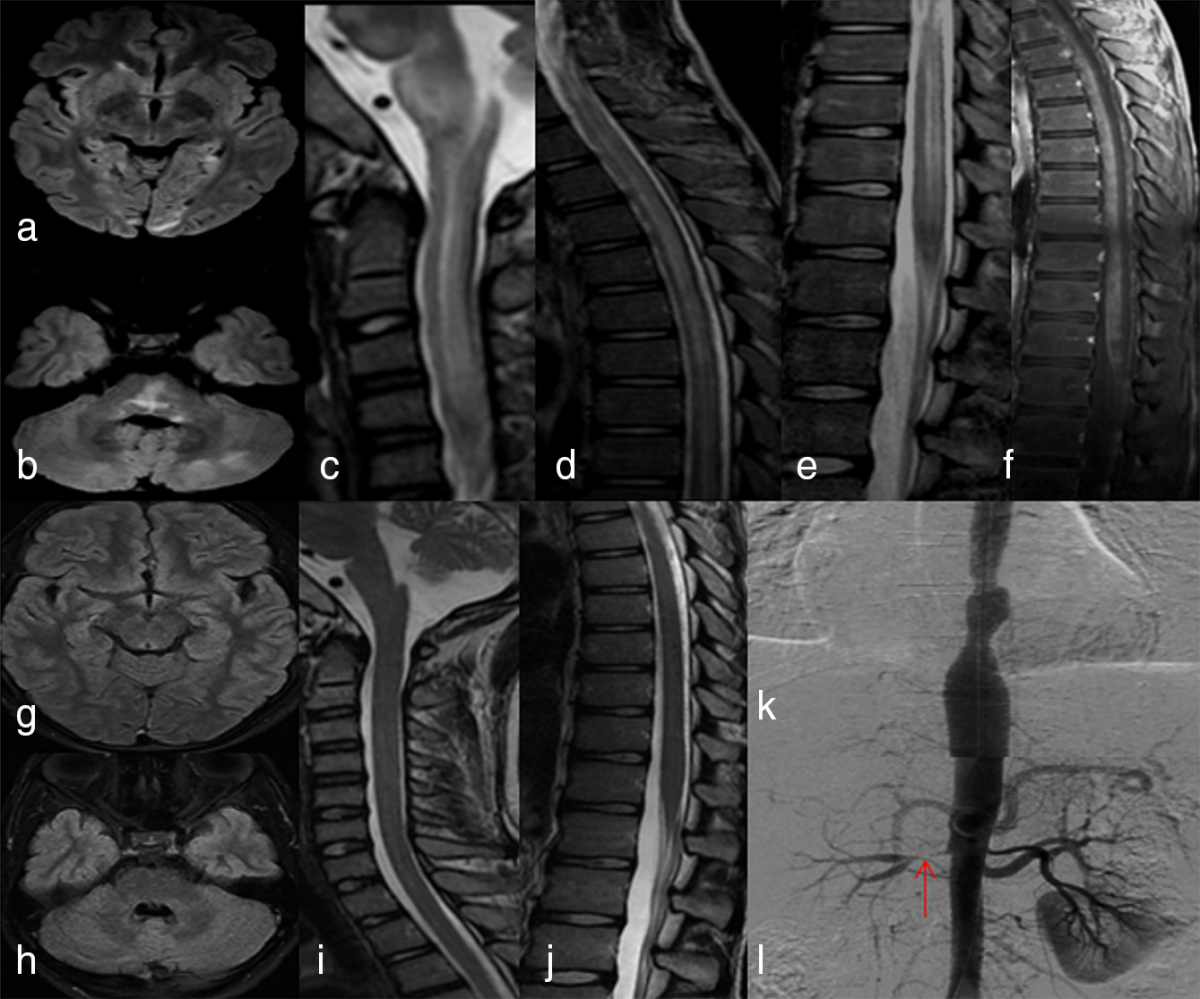
Fluid-attenuated inversion recovery images (a,b) of the brain show signal changes in the bilateral occipital subcortical white matter, pons, medulla and left cerebellum. Intramedullary *T*_2_ hyperintensity with mild cord expansion (c–e) is seen in the entire spinal cord with extensive leptomeningeal enhancement on the post-contrast study (f). Angiogram (k,l) shows right proximal renal artery stenosis (red arrow in l) and focal area of stenosis in the descending aorta. An MRI repeated after 4 weeks shows complete regression of the signal changes in the brain and the spinal cord (g–j).

An MRI (Figure 1) showed multifocal *T*_2_/fluid-attenuated inversion recovery hyperintensities involving the bilateral putamen, subcortical white matter in bilateral occipital lobes, pons and medulla. Long segment *T*_2_ hyperintensity with minimal cord expansion was noted involving the cervicodorsal spinal cord. A post-gadolinium study showed punctate enhancement in the brainstem and striking, diffuse leptomeningeal enhancement extending from the prepontine region to the entire spinal cord. The cerebrospinal fluid (CSF) examination was normal. Evaluation for hypertension revealed aortoarteritis involving the descending thoracic aorta with renal artery stenosis.

## Differential diagnosis

Based on the clinical presentation and the imaging findings, the differential diagnoses entertained initially were, PRES, tubercular meningitis and neuromyelitis optica (NMO). The extensive contrast enhancement on MRI and common occurrence with varied clinical presentation in this part of world made us consider tubercular meningitis as a differential diagnosis. However, it was negated by a normal CSF examination. No clinical evidence of myelopathy argued against NMO. Overall, the classical symptoms (headache, seizure and visual blurring), disproportionate imaging findings and minimal abnormalities on neurological examination in the background of accelerated hypertension in a young patient favoured the diagnosis of PRES.

## Treatment

The patient was treated based on the diagnosis of PRES. Symptomatic treatment for seizures and headache was given, with proper control of hypertension. Definitive treatment for renal artery stenosis was planned.

## Outcome and follow-up

A repeat MRI after 28 days revealed complete regression of all signal changes in the brain and spinal cord with decrease in cord swelling earlier seen on MRI, thereby confirming the diagnosis of PRES.

## Discussion

Classically, PRES is associated with hypertension, eclampsia and cyclosporine toxicity, although the list of causes have grown with time.^1^^–^^3^ Patients usually present with seizures, headache, vision change, altered sensorium or focal neurological deficits, which can be acute or progressive over days.^4^ Symmetric signal change in the parieto-occipital white matter is seen on MRI in majority of cases, which are reversible when the inciting cause is removed. Many theories have been proposed to explain the pathogenesis; however, the loss of autoregulation and endothelial dysfunction leading to vasogenic edema is the most widely speculated theory.^5^ In about 75% cases of PRES, moderate-to-severe hypertension is present, although the mean blood pressure is never above the autoregulatory limits of 150–160 mmHg in most cases and there is no relation between the severity of clinical and imaging findings with the degree of hypertension.^6^ Bartynski et al^6^ reviewed the MRI imaging of 136 cases of PRES and described three common hemispheric patterns of involvement: holohemispheric, parietal–occipital and superior frontal sulcal patterns, with overall parietal–occipital involvement found in 98% of their cases. These typical patterns can have variable expression or asymmetry in an individual patient. They also found involvement of the cerebellum, brainstem, temporal lobes, splenium and basal ganglia in atypical cases. Isolated involvement of these structures without parietal–occipital involvement is, however, uncommon. Spinal cord involvement was not described by Bartynski.^6^ We found only eight cases of PRES with spinal cord involvement reported so far. de Havenon et al,^7^ while reporting on two cases of PRES with spinal cord involvement, reviewed six others from the literature and proposed a new syndrome “PRES with spinal cord involvement”.

Our case is unique in many aspects. Extensive cord signal change in the absence of clinical myelopathy is hard to explain. It may represent vasogenic oedema, in which case, an extension of the theory of autoregulatory failure in PRES may be entertained. Post-contrast enhancement, as seen in our case, is rarely reported in the literature. The reason might be that in most cases of PRES, given the typical imaging findings, a contrast examination is rarely performed. Anyway, this finding may suggest a role for leaky endothelium in the pathogenesis of PRES. This aspect needs to be further investigated. The cause of hypertension in our case was found to be aortoarteritis, leading to renal artery stenosis. Karande et al^8^ reported PRES associated with Takayasu arteritis in a 10-year-old male. No spinal cord abnormality was reported in their case. Aortoarteritis *per* *se* causing cord signal changes seems untenable, as the reversal of findings after treatment of hypertension excludes any vascular (ischaemic) cause.

## Learning points

1. Spinal cord signal alterations can occur in PRES without clinical myelopathy. Familiarity with this rare imaging finding may help avoid unnecessary investigations and inappropriate treatment in PRES.
2. Post-contrast enhancement, as seen in our case, may point towards a role for leaky endothelium in the pathogenesis of PRES.

## References

[1] Waldron RL 2nd, Abbott DC, Vellody D Computed tomography in preeclampsia-eclampsia syndrome. AJNR Am J Neuroradiol 1985; 6: 442–3. 3923804 PMC8335350

[2] Weingarten K, Barbut D, Filippi CG, Filippi C, Zimmerman RD Acute hypertensive encephalopathy: findings on spin-echo and gradient-echo MR imaging. AJR Am J Roentgenol 1994; 162: 665–70. 8109519 10.2214/ajr.162.3.8109519

[3] Truwit CL, Denaro CP, Lake JR, DeMarco T MR imaging of reversible cyclosporin A-induced neurotoxicity. AJNR Am J Neuroradiol 1991; 12: 651–9. 1882738 PMC8331570

[4] Garg RK Posterior leukoencephalopathy syndrome. Postgrad Med J 2001; 77: 24–8. 11123390 10.1136/pmj.77.903.24 PMC1741870

[5] Strandgaard S, Olesen J, Skinhoj E, Lassen NA Autoregulation of brain circulation in severe arterial hypertension. Br Med J 1973; 1: 507–10. 4692673 10.1136/bmj.1.5852.507 PMC1588676

[6] Bartynski WS, Boardman JF Distinct imaging patterns and lesion distribution in posterior reversible encephalopathy syndrome. AJNR Am J Neuroradiol 2007; 28: 1320–7. 17698535 10.3174/ajnr.A0549 PMC7977645

[7] de Havenon A, Joos Z, Longenecker L, Shah L, Ansari S, Digre K Posterior reversible encephalopathy syndrome with spinal cord involvement. Neurology 2014; 83: 2002–6. 25355822 10.1212/WNL.0000000000001026

[8] Karande S, Jagtap S, Joshi A Posterior reversible encephalopathy syndrome revealing Takayasu's arteritis. Indian J Pediatr 2009; 76: 218–20. 19082534 10.1007/s12098-008-0224-1

